# Pain Prevalence and Management in an Internal Medicine Setting in Italy

**DOI:** 10.1155/2014/628284

**Published:** 2014-01-20

**Authors:** Fabio Fabbian, Alfredo De Giorgi, Marco Pala, Alessandra Mallozzi Menegatti, Massimo Gallerani, Roberto Manfredini

**Affiliations:** Department of Internal Medicine, St. Anna General Hospital, Via A. Moro 8, 44124 Ferrara, Italy

## Abstract

*Background*. Since data on pain evaluation and management in patients admitted to internal medicine wards (IMWs) are limited, we aimed to evaluate these aspects in a cohort of internistic patients. *Methods*. We considered all patients consecutively admitted from June to December 2011 to our unit. Age, gender, and length-of-hospital-stay (LOS) were recorded. Comorbidities were arbitrarily defined, and pain severity was evaluated by Numeric Rating Scale (NRS) on admission and discharge. *Results*. The final sample consisted of 526 patients (mean age 74 ± 14 years; 308 women). Significant pain (NRS ≥ 3) was detected in 63% of cases, and severe (NRS ≥ 7) in 7.6%. Pain was successfully treated, and NRS decreased from 4.65 ± 2.05 to 0.89 ± 1.3 (*P* < 0.001). Compared with subjects with NRS < 3, those with significant pain were older (75.5 ± 13.9 versus 72.9 ± 14.5 years, *P* = 0.038), and had a higher LOS (7.9 ± 6.1 versus 7.3 ± 6.8, *P* = 0.048). Significant pain was independently associated with age (OR 0.984, *P* = 0.018), cancer (OR 3.347, *P* < 0.001), musculoskeletal disease (OR 3.054, *P* < 0.0001), biliary disease (OR 3.100, *P* < 0.01), and bowel disease (OR 3.100, *P* < 0.003). *Conclusion*. In an internal medicine setting, multiple diseases represent significant cause of pain. Prompt pain evaluation and management should be performed as soon as possible, in order to avoid patients' suffering and reduce the need of hospital stay.

## 1. Introduction

Pain is a common symptom and moderate-to-severe pain has been reported to affect up to 50% of community dwelling older adults and up to 80% of nursing home residents [1]. In Italy, since March 2010, a complete report of assessment of pain in clinical records described as type, measurement, treatment, and degree of relief became compulsory by law n° 8 “Provision aimed at ensuring access to palliative care and pain therapy.” Comorbidity is actually the main problem that physicians have to deal with, especially in internal medicine wards (IMWs) [2], due to mean age of patients and multiple-organ dysfunction.

A survey analyzing the quality of documentation related to pain measurement and treatment in patients discharged from hospitals of the Tuscany Region of Italy has been recently published [3]. Out of 2,459 subjects investigated, the majority were aged 70 to 79 years, and 63.77% reported medical Diagnosed Related Groups (DRGs), mainly cardiovascular diseases. These data defined pain as a very frequent compliant in hospital settings. The great majority of papers published on pain management are focused on disease-specific conditions, whereas data describing pain management in patients admitted in IMWs are very limited. Measurement of pain is based on its quantification using ordinal or category scales that offer patients a simple method to evaluate intensity of pain. Numeric Rating Scale (NRS) is a simple tool, where the patient is only required to choose a numerical value indicating the intensity of pain.

The aim of this study was to evaluate pain prevalence and management associated with a simple pain measured by NRS, in a cohort of consecutive patients admitted to an IMW.

## 2. Materials and Methods

We conducted an observational prospective study involving all adult patients consecutively admitted from 1 June to 31 December 2011 to the 30 beds IMW of Clinica Medica, Azienda Ospedaliera-Universitaria S.Anna, Ferrara, Italy. All patients received detailed information about the study and gave consent to participate. Subjects with cognitive impairments, with major sensorial deficits, or unable to understand the opposite information paper, and those who refused to participate were excluded. The study was approved by the local ethical committee (no. 119-2011; 27/10/2011).

Age and gender were recorded, and length-of-hospital-stay (LOS) was calculated. Comorbidity was arbitrarily defined by classifying diseases in subgroups: cancer, heart, pulmonary, vascular, musculoskeletal, neurological, cutaneous, renal, hepatic, biliary, metabolic, pancreatic, gastric, and bowel diseases. Moreover, the presence of positive history of surgery was also evaluated.

Oncologic disease included malignancy in every apparatus or organ. Cardiac disease included infectious, inflammatory, ischaemic, and valvular diseases and arrhythmias. Heart failure was excluded. Pulmonary diseases included infectious, inflammatory, and vascular disease of lungs or pleura. Vascular diseases included damage of main and medium venous or arterial vessels. Musculoskeletal diseases included all the processes leading to altered function of bone and muscles. Neurological diseases included degenerative and ischaemic damage of central nervous system. Cutaneous diseases included all processes that altered the skin. Nephrologic diseases included all processes responsible of acute or chronic reduction of renal function. Hepatic diseases included all processes producing liver dysfunction. Biliary diseases included infectious, inflammatory, and dysplastic processes with and without jaundice and gallstones. Metabolic diseases included obesity and diabetes mellitus. Pancreatic diseases included acute and chronic pancreatitis and malignancy. Gastric diseases included infectious, inflammatory, and dysplastic processes. Bowel diseases included infectious, inflammatory, and dysplastic processes. Diverticulitis were also included. Postsurgery condition included all patients who underwent any recent operation in any apparatus or organ. To evaluate comorbidities, the Charlson comorbidity index was calculated [4]. Such score is calculated on the basis of age and the presence or absence of the following conditions: HIV infection, cerebrovascular disease, chronic pulmonary disease, congestive heart failure, connective tissue disease, dementia, hemiplegia, leukemia, malignant lymphoma, myocardial infarction, peripheral vascular disease, ulcer disease, diabetes mellitus with or without organ damage, liver and renal disease classified as mild, moderate and severe, and metastatic and nonmetastatic malignant solid tumor. Moreover, data indicating location and distribution, duration and periodicity, and quality of pain were also recorded.

NRS was used in order to assess pain. Patients defined their pain referring to a 0 to 10 scale with “0” representing “no pain at all” and “10” representing “the worst imaginable pain.” Pain was defined as mild (1 ≤ NRS < 3), moderate (3 ≤ NRS < 7), and severe (NRS ≥ 7). Subjects were considered to have significant pain when NRS was equal or greater than 3. NRS was recorded on both admission and discharge, and administration of analgesic drugs before and during admission was also recorded.

Data were analyzed using SPSS (SPSS Inc., Chicago, IL); continuous data were reported as mean and standard deviation, and categorical variables as percentage. Patients were divided into two groups: patients without pain and with mild pain (NRS < 3) and patients with moderate to severe pain (NRS ≥ 3). These two groups were compared using *t*-test, Mann Whitney *U* test, and Chi Squared tests, as appropriate. A two-tailed *P* value of <0.05 was considered significant. In order to evaluate which variables were independently associated with moderate and severe pain, logistic regression analysis was performed.

## 3. Results

The final sample consisted of 524 patients (mean age 74 ± 14), 307 women and 217 men. LOS was 7.7 ± 6.4 days. Classification and frequency of comorbidities and prevalence of the different intensity of pain patients groups *on admission* are indicated in Table 1. Moderate and severe pain was detected in 218 cases (41.6%), 106 of whom (48.6%) were treated with analgesic drugs before admission. NRS = 0 was detected in 195 subjects (37.2%). Pain was defined as severe (NRS ≥ 7) in 40 subjects (7.6%).

**Figure 1:**
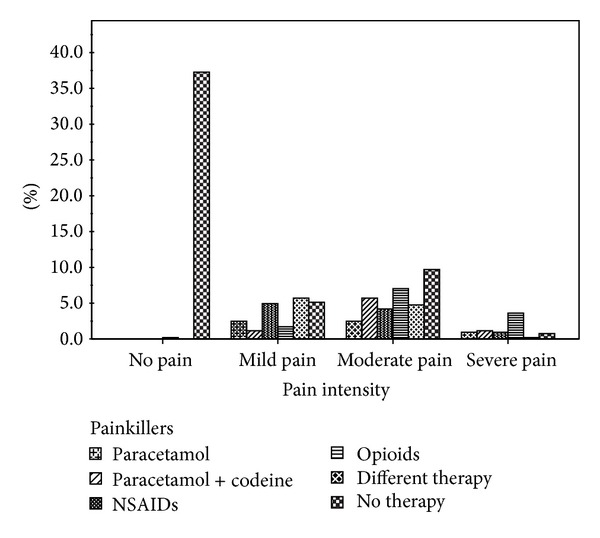
Relationship between pain intensity and treatment (in the course of hospitalization).

Pain was defined as visceral and continuous in 213 patients (40.6%), burning in 146 (27.9%), cramping in 113 (21.6%), oppressive in 82 (15.6%), and lancinating in 20 (3.8%). Pain duration was reported to be less than 2 hours in 9 cases (1.7%), between 2 and 6 hours in 47 cases (9%), between 6 and 12 hours in 77 cases (14.7%), between 12 hours and 3 days in 70 cases (13.4%), and more than 3 days in 128 cases (24.4%). Patients were treated with NSAIDs in 61 cases (11.6%), paracetamol in 31 cases (5.9%), paracetamol plus codeine in 42 cases (8%), opioids in 75 cases (14.3%); 84 subjects (16%) were treated with different drugs. In Figure 1, treatment of pain during hospitalization in the different NRS groups is reported. At discharge NRS was 0.5 ± 1.1, significantly lower than the mean value recorded at admission (mean reduction: 2.3 ± 2.4). Moreover, in patients with significant pain, NRS decreased from 4.65 ± 2.05 to 0.89 ± 1.3 (*P* < 0.001). Subjects with significant pain were older, had greater prevalence of cancer, heart, peripheral vascular, musculoskeletal, biliary, and bowel diseases, and had also higher prevalence of surgery history compared with patients with NRS < 3 (Table 2). Again, LOS was longer in patients with significant pain than in those without (7.9 ± 6.1 versus 7.3 ± 6.8, *P* = 0.048) (Table 2). Significant pain was independently associated with age (OR 0.984, 95% CI 0.971–0.997, and *P* = 0.018), cancer (OR 3.347, 95% CI 1.952–5.739, and *P* < 0.001), musculoskeletal disease (OR 3.054, 95% CI 1.683–5.541, and *P* < 0.0001), biliary disease (OR 3.100, 95% CI 1.312–7.328, and *P* < 0.01), and bowel disease (OR 3.100 95% CI 1.482–6.482, and *P* < 0.003), whereas all the other clinical characteristics were not associated to significant pain.

## 4. Discussion

This study shows that pain (moderate to severe) is present in more than 40% of patients admitted to an IMW, and attention and appropriate management may lead to a significant pain reduction. Moreover, such successful outcome might have also economic advantage, derived from a significant reduction of LOS. In 2012, Gustavsson et al. [5] evaluated the costs of diagnosis related to chronic pain in 837,896 patients living in a geographical region of Sweden with 1.56 million inhabitants. The mean total costs of all patients, both direct and indirect, were 6,400 EUR per patient in the year, of which 14% were inpatient care costs. Interestingly, only 79 EUR (<2%) were the cost of analgesic drugs.

Nowadays, pain is considered not merely as a symptom but as an actual disease process [6], and for this reason, prevalence of pain could be only high in patients admitted in IMWs. Nevertheless, although the use of specific scale for its evaluation appears to be an important mean for its treatment, physicians do not evaluate pain sufficiently. In 2011, Haller et al. [7] evaluated the implementation of a collaborative quality improvement program enrolling patients discharged from a teaching hospital of 2,096 beds. They concluded that the program improved both pain management and pain relief. A similar attitude was the reason for conceiving the law number 38, 15th March 2010. Diseases inducing pain are well known by internists that should evaluate the condition as soon as possible, in order to avoid patients' suffering and indirectly to decrease LOS. Different attitudes influence physicians' approach to pain and religion may affect the evaluation of prevalence of pain as well. Kaldjian et al. [8] showed that, among internists, being less or more aggressive in the support for terminal sedation depends on religious service attendance. On the other hand, surgeons are used not to treat abdominal pain in order to correctly diagnose the degree of organ damage [9], even if opioids do not aggravate clinical conditions in subjects with abdominal pain [10].

Pain is a very important medical and social problem worldwide. In 2006 Breivik et al. [11] reported that 19% of adult Europeans suffered pain for six month. The previous studies conducted in Italy aimed to detect the prevalence of pain in many hospitals or different hospital wards. Gianni et al. [12] evaluated the prevalence of pain in Italian geriatric hospital departments, and found that pain was present in 63.7% of patients. Such percentage is higher than that found in our study. In fact, we found that age was independently related to significant pain and age of patients admitted to geriatric ward is higher than the age of patients admitted to IMW. Gianni et al. reported a high prevalence of comorbidities, and pain was undertreated: only 49% of subjects received treatment, of whom only 24.5% successfully. Costantini et al. [13] quantified the prevalence of pain among hospitalized Italian patients older than 18 years of the Liguria region. Out of 4,709 inpatients, 56.6% suffered pain during the last 24 hours. In the logistic regression analysis, gender, diagnosis, and days from surgery were significantly associated with increased pain prevalence. In a cancer population of 258 patients hospitalized for at least 24 hours, pain was detected in 51.5% of cases, and it was ascribed to surgery in 49.6% and to the tumor mass itself in 29.3% of patients [14]. Visentin et al. [15] performed a survey analysing 4,523 inpatients throughout Italy, 91.2% of them reported pain, that was evaluated as severe in 46.6%. The prevalence of severe pain was significantly lower in women and was double in general medicine wards compared to surgical wards. The authors reported that only 28.5% of the population had been treated with analgesics in the past 24 hours. In our study, 48.6% of patients received analgesics drugs before admission even if the probability of receiving painkillers was lower for general medicine wards than for surgical wards [15]. Melotti et al. [16] investigated 892 patients and found that prevalence of pain was high among young adults or divorced/separated individuals. Allione et al. [17] analyzed the relationship between administration on analgesic and triage priority score in 393 subjects admitted in an emergency department in North-West of Italy. Similarly to our study, the majority of them had a pain duration greater than 12 hours, besides among subjects with severe pain 51% received analgesic, among those with moderate pain 31% received analgesic and among those with mild pain only 20% received analgesics, and the great majority of them were treated with acetaminophen and anti-inflammatory drugs. The authors concluded that underuse of analgesic in their emergency department was a problem. Comorbidities could represent another obstacle in treating pain in IMWs. Patients admitted to IMWs are old and affected by different chronic diseases, and multiple medications are needed. Adding painkillers to a long list of drugs in these patients provides the potential for substance interaction and an increase in adverse events. However, comorbidities themselves could be the cause of pain, in our study the number comorbidities of patients was high and our arbitrary classification of them was better related to pain than the Charlson score.

To the best of our knowledge, this is the first study that analysed all the aspects of management of pain in an Italian IMW after the introduction of the 2010 Italian specific law. However, some limitations should be underlined. First of all we did not include in our analysis pain management in the emergency department; in fact, drugs given to patients at the time of that evaluation could influence pain evaluation at the time of IMW admission. Second, we did not screen subjects with cognitive impairment, these kind of evaluation is better performed in geriatric wards where physicians are trained to manage such a patients. Third, we include in the analysis postoperative patients; however, our aim was merely do analyse pain management in an IMW, where due to shortage of hospital beds it could be that postoperative patients need to be managed. Fourth, we measured pain by ordinal scale on admission and discharge, and we have not intermediate data. On the other hand a reduction in the values of pain scale and administration of drugs for pain management underlines a careful evaluation of this parameter. Fifth, we arbitrarily defined patients without significant pain those with NRS < 3, due to the fact that such pain intensity does not require medical intervention.

An appropriate knowledge of prevalence of pain, its causes and intensity, and patient satisfaction after pain management are key points for evaluating the impact of the interventions performed and the overall skill of our teams to tackle pain emergency in the hospitalized population. In our study, pain was successfully treated with standard therapy, even if moderate to severe pain was related to higher LOS, with consequent time and resources consuming. Our data showed that pain management could be considered good, and mean NSR value on discharge could be considered satisfactory. However, the use of scale for determining intensity of pain could not be completely satisfactory.

In conclusion, pain is a frequent condition in subjects admitted in IMWs, and multiple diseases other than cancer represent possible causes. Since internists are familiar with these kind of diseases, a prompt pain evaluation and management should be performed as soon as possible, in order to either avoid patients' suffering and reduce the length of hospital stay.

## Figures and Tables

**Table 1:** Classification, frequency of underlying comorbidities, and prevalence of different intensity of pain, on admission.

Comorbidities	Number of cases (%)
Cancer disease	109 (21%)
Heart disease	175 (33%)
Pulmonary disease	123 (23%)
Vascular disease	128 (24%)
Musculoskeletal disease	86 (16%)
Neurological disease	95 (18%)
Cutaneous disease	20 (4%)
Renal disease	77 (15%)
Metabolic disease	85 (16%)
Hepatic disease	55 (10%)
Biliary disease	47 (9%)
Pancreatic disease	16 (3%)
Gastric disease	63 (12%)
Bowel disease	56 (11%)
Recent surgery history	42 (8%)

No pain	195 (37.2%)
Mild pain	111 (21.2%)
Moderate pain	178 (34%)
Severe pain	40 (7.6%)

**Table 2:** Clinical parameters, Charlson index score, comorbidities, mean duration of hospital length-of-stay (LOS), and Numeric Rating Scale (NRS) recorded on admission and discharge in patients with and without significant pain.

	NRS < 3	NRS ≥ 3	*P*
Number of cases (%)	195 (37.2)	329 (62.8)	
Age (years)	75.5 ± 13.9	72.9 ± 14.5	0.038
LOS (days)	7.3 ± 6.8	7.9 ± 6.1	0.048
Charlson index	5.27 ± 3.0	6.21 ± 4.1	NS
NRS at admission	0.04 ± 0.4	4.65 ± 2.05	<0.001
NRS at discharge	0.01 ± 0.14	0.89 ± 1.3	<0.001
Reduction in NRS during admission	0.03 ± 0.43	3.76 ± 2.11	<0.001
Cancer disease	20 (10.2%)	89 (26.8%)	<0.0001
Heart disease	53 (27.1%)	122 (36.8%)	0.027
Pulmonary disease	38 (19.5%)	85 (25.6%)	NS
Vascular disease	32 (16.4%)	96 (29%)	0.001
Musculoskeletal disease	16 (8.2%)	70 (21.1%)	<0.001
Neurological disease	30 (15.2%)	65 (19.6%)	NS
Cutaneous disease	3 (1.5%)	17 (5.1%)	NS
Renal disease	22 (11.2%)	55 (16.6%)	NS
Metabolic disease	14 (7.1%)	41 (12.3%)	NS
Hepatic disease	7 (3.5%)	40 (12%)	0.001
Biliary disease	17 (8.7%)	80 (24.1%)	<0.001
Pancreatic disease	4 (2%)	12 (3.6%)	NS
Gastric disease	13 (6.6%)	50 (15.1%)	0.003
Bowel disease	10 (5.1%)	46 (13.8%)	0.001
Recent surgery history	6 (3%)	36 (10.8%)	0.001
